# A Throat that Clicks

**DOI:** 10.22038/ijorl.2021.51303.2752

**Published:** 2021-07

**Authors:** Kuganathan Ramasamy, Jeyasakthy Saniasiaya

**Affiliations:** 1 *Department of Otorhinolaryngology, Hospital Tuanku Ja’afar, Jalan Rasah, 70300 Seremban, Negeri Sembilan, Malaysia.*; 2 *Department of Otorhinolaryngology, Faculty of Medicine, University of Malaya, Jalan Universiti, 50603 Kuala Lumpur, Wilayah Persekutuan Kuala Lumpur, Malaysia.*

**Keywords:** Clicking, Hyoid, Larynx, Throat

## Abstract

**Introduction:**

Clicking larynx syndrome is a rare condition that may be intriguing to the attending clinician. Patients typically present with clicking sensations in the neck, often obvious during head movement or swallowing. Due to the scarce presentation of such cases, clicking larynx syndrome harbors a high propensity to be an overlooked diagnosis, resulting in a clinical stalemate.

**Case Report:**

Herein, we present a case of clicking larynx in a young girl followed by an overview of the latest literature on the aetiology and treatment options. This case aims to reinforce the presence of this entity further and subsequently increase its awareness among clinicians.

**Conclusion:**

Expeditious diagnosis is imperative not just for the eventual treatment but also for timely relief to the anxious patients who would have been perplexed by the strange clicking in the throat.

## Introduction

Clicking larynx is an enigmatic entity that often perplexes clinicians. Albeit rare, with less than twenty cases reported in the literature, it is often attributed to hyoid or thyroid cartilage anatomical abnormalities. It typically presents with a clicking sound in the neck, more pronounced during swallowing or head movement. In exceptional situations, the strange clicks may cause psychological distress and social stigma to the patient. To date, surgical treatment has proven to be successful, and it is tailored according to patients’ findings.

## Case Report

**Fig 1:**
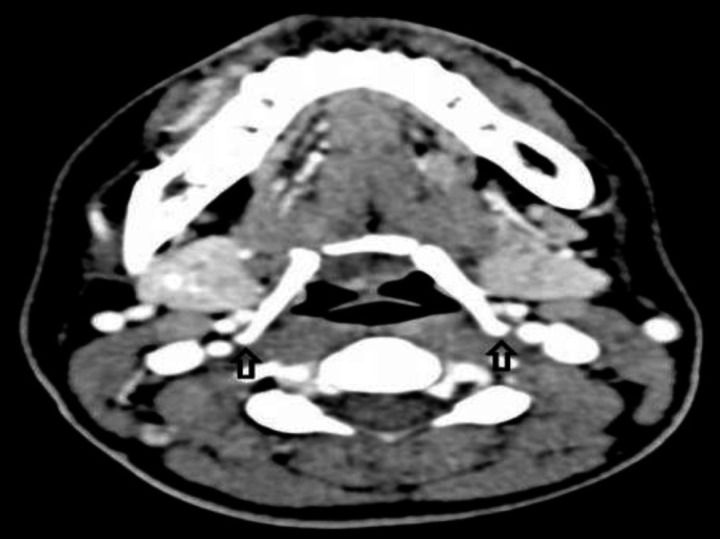
Computed tomography revealed bulky greater horns of the hyoid bone which is in close proximity with body of fourth cervical vertebrae

A previously healthy 21-year-old female presented with painless clicking sounds in the throat for the past two years. There were no inciting factors that led to the sound. Clicking sounds occurred initially on random occasions with no provoking factors, which later became more frequent and bothersome. She denied any dysphagia, odynophagia, or dysphonia. Examination revealed no abnormalities in the neck. However, the clicking sound was noted to be more pronounced with slight lateral pressure and manipulation of the hyoid bone. Flexible fibreoptic endoscopy and barium swallow study showed no abnormalities. Computed tomography (CT) neck revealed bulky greater horns of the hyoid bone, which is in close proximity with the body of the fourth cervical vertebrae as well as the internal carotid artery (Figure 1). The findings and surgical treatment choice were then explained to the patient. Albeit opting for the conservative option, she was nevertheless relieved upon learning the cause of her symptoms and is to date on regular follow-up.

## Discussion

Clicking sound in the larynx is a perplexing condition that might cause significant distress to the affected patients. It is a scarce entity with less than twenty cases reported to date (1). Heuveling et al., in their literature review, mentioned that majority of the cases involves female with a substantial number less than 30 years old which is similar to our patient.

Amongst the myriad aetiologies reported, notable ones include impingement on the cervical spine by the elongated or bulky greater horn of hyoid and friction between medially displaced superior cornu of thyroid cartilage with hyoid bone (2-5). Other causes include shortened thyrohyoid distance and abnormal bone formation in the thyrohyoid ligament. In our case, the causative factor was bulky greater horns of the hyoid, thus establishing this factor as a predominant cause for clicking larynx syndrome.

The pathophysiology of this rare entity remains a conundrum. Various predisposing factors have been brought forward, albeit with unsatisfactory evidence. Among those postulated factors include a change in the shape of thyroid cartilage or an increase in laxity of the joint ligaments with aging. However, this fact lacks credence as the majority of the patients reported previously are young adults and adolescents. Similarly, neck trauma causing alteration to laryngeal anatomy was proposed, and this too was only present in a minority of the patients (1). Other predisposing factors mentioned in the literature include mandibular advancement surgeries, resection of the styloid process, and mouth breathing (1). 

Albeit its rarity and queer presentation, a thorough and meticulous clinical approach would always ensure proper establishment of this diagnosis and help to differentiate from other hyoid-associated pathology such as hyoid bone syndrome. The latter habitually present with localized pain over the hyoid region (6). Neck examination should involve an attempt to reproduce the clicking via palpation and manipulation of the hyoid bone, particularly with action with laryngeal movements such as swallowing. This will be able to shed some light on the possible underlying mechanism. Flexible laryngoscopy can reveal abnormalities such as medially displaced superior cornu of the thyroid cartilage. Nevertheless, CT neck remains the gold standard modality to reveal the possible mechanism behind the clicking larynx, besides for surgical planning. In cases of shortened thyrohyoid distance, barium swallow may additionally demonstrate overriding of the thyroid cartilage over the hyoid bone.

Countless studies exhort surgery as the sole treatment, as all the operated patients showed complete resolution of symptoms without any complications (1). There has been no other alternative treatment being reported. As for the type of surgery, it depends on the anatomical deformity causing the clicking sound, which includes resection of either the enlarged greater horn of hyoid, displaced superior cornu of the thyroid cartilage, the upper part of thyroid lamina, or abnormal bone formation in the thyrohyoid ligament (1-5). Having said that, a conservative approach opted in our case as the patient felt reassured of the benign nature of the entity and was not keen on surgery.

## Conclusion

Notwithstanding its rarity, clicking larynx syndrome can be a very puzzling finding to the uninitiated and, in exceptional situations, may even cause psychological distress and social stigma to the patient. Understanding possible aetiology and treatment options are of paramount importance towards the resolution of the disease. Surgical management is proven to be a success in most cases. However, reassurance of its benign nature can provide huge relief to patients who may opt for a conservative approach. This case report intends to further establish this rare syndrome and raise awareness among physicians regarding this entity. 
